# Analysis of factors influencing rapid treatment initiation decisions among people living with HIV: focusing on the role of social support

**DOI:** 10.3389/fpubh.2026.1758402

**Published:** 2026-02-02

**Authors:** Yujie Wu, Jinyu Wang, Yuting Li, Fei Wang, Sheng Li

**Affiliations:** 1 School of Public Health, Gansu University of Chinese Medicine, Lanzhou, China; 2 School of Public Health, Lanzhou University, Lanzhou, China; 3 First Clinical Medical College, Gansu University of Chinese Medicine, Lanzhou, China; 4 The No.2 People’s Hospital of Lanzhou, Lanzhou, China

**Keywords:** antiretroviral therapy initiation, people living with HIV/AIDS (PLWHA), rapid initiation of antiretroviral therapy, social support, treatment decision-making

## Abstract

**Background:**

To investigate the impact of social support factors on the acceptance of rapid initiation of antiretroviral therapy (ART) among people living with HIV/AIDS (PLWHA), and to provide evidence for developing personalized interventions to improve the rapid ART initiation rate.

**Methods:**

A cross-sectional study was conducted among patients visiting Lanzhou Pulmonary Hospital between September 2024 and January 2025. Data were collected via questionnaires. Variable selection was performed using univariable logistic regression; those with a significance level of *p* < 0.05 were included in a multivariable logistic regression model. Group differences in rapid initiation rates were assessed using the chi-square test.

**Results:**

Among the 530 participants (506 males, 481 Han ethnicity), the highest proportion was in the 31 to <46 age group. Univariable analysis showed that age, education, cross-regional treatment, marital status, monthly income, disclosure to spouses/family, and having infected peers were associated with rapid ART initiation. The multivariable model identified that younger age groups (15- < 31 and 31- < 46), cross-district treatment within the city, monthly income ≥5,000 CNY, disclosure to spouses/family, and having infected peers were facilitators, while being unmarried was a barrier. Rapid initiation rates differed significantly based on spouses’/family members’ attitudes post-disclosure (*χ*^2^ = 4.281, *p* = 0.039) and provision of support (*χ*^2^ = 4.281, *p* = 0.039), but not on peer support provision (*p* = 0.058). Among the support provided by spouses/family members, psychological support was the most common and was associated with the highest rapid ART initiation rate. The proportions of financial support and material support were similar, and their corresponding rapid initiation rates were also comparable. PLWHA who received no support had the lowest rapid initiation rate, which was significantly lower than rates observed with any form of support. Similar results were observed in the subgroup with infected peers: psychological support had the highest proportion and the greatest rapid ART initiation rate, while the absence of peer support was associated with the lowest proportion and the smallest rapid initiation rate.

**Conclusion:**

Integrating social factors into rapid ART initiation interventions, through a support network connecting families, peers, and healthcare institutions, can enhance treatment willingness and timeliness, ultimately improving outcomes for PLWHA.

## 1 Introduction

Acquired Immune Deficiency Syndrome (AIDS) is an immunodeficiency syndrome caused by infection with the Human Immunodeficiency Virus (HIV). Due to its high infectivity, AIDS has become a major global public health challenge. Antiretroviral therapy (ART) is currently the most effective treatment, capable of effectively suppressing viral replication and significantly improving patients’ immune function (1, 2).

In recent years, studies both domestically and internationally have consistently demonstrated that rapid initiation of ART helps improve treatment outcomes. These benefits include shortening the viral suppression time for PLWHA, reducing the treatment dropout rate, and lowering the risk of complications (3–5). This has significant public health implications for HIV prevention. However, despite strong recommendations from various studies and guidelines for rapid ART initiation, improving the rapid initiation rate still faces many challenges in practice. For example, after initial diagnosis, PLWHA may experience psychological fear of long-term medication, lack knowledge about the disease, or fail to receive timely ART due to factors such as inconvenient transportation and insufficient financial support. These factors highlight the urgency of further optimizing practical measures to promote rapid ART initiation among PLWHA (6).

In addition to the influence of basic demographic factors of PLWHA, social support is also recognized as a significant factor affecting their treatment decisions. Social support includes material, emotional, and informational assistance provided by family, friends, peers, and healthcare providers. It is widely considered a key determinant of health behaviors in patients with chronic diseases (7). After diagnosis, PLWHA often experience substantial psychological impact, including fear and anxiety about the unknown disease. A strong social support network can enhance their psychological resilience and increase their confidence and motivation to cope with the condition.

Although numerous previous studies have investigated factors influencing treatment decisions among PLWHA, most have focused on individual-related factors. Few studies have explored the impact of social support on treatment decisions, and limited literature provides detailed elaboration on the sources and categories of social support. Therefore, this study focuses on analyzing the influence of social support on treatment decisions among PLWHA. It specifically examines the role of social support in shaping their decisions and identifies key sources and types of social support. The findings aim to provide targeted practical evidence for developing clinical interventions and related prevention policies.

## 2 Materials and methods

### 2.1 Study population

A cross-sectional survey was conducted among patients who visited Lanzhou Pulmonary Hospital (Gansu Provincial HIV Antiviral Treatment Quality Control Center) between September 2024 and January 2025. The inclusion criteria were as follows: ① age ≥ 15 years; ② voluntary provision of informed consent; ③ ability to read and complete the questionnaire independently; and ④ currently receiving regular ART. Patients were excluded if they ① had severe psychiatric disorders or physical disabilities that prevented them from cooperating with the survey. This study was approved by the Ethics Committee of Lanzhou Pulmonary Hospital (**Approval No. 2024050601**).

### 2.2 Methods

A convenience sampling method was used to recruit patients who visited Lanzhou Pulmonary Hospital (Gansu Provincial HIV Antiviral Treatment Quality Control Center) between September 2024 and January 2025. Following a literature review and expert consultation, the “Questionnaire on Influencing Factors of Rapid Antiretroviral Therapy” (8) was selected and employed. Trained investigators used the “Questionnaire Star” online platform to invite patients waiting for outpatient consultations to voluntarily scan a QR code and participate in the survey. The sample size was calculated by integrating the objective of this study with findings from previous research, using the following formula:


$$N=\frac{{\left(Z_{1-\alpha /2}\right)}^{2}(1-P)}{\varepsilon^{2}P}$$

The required sample size (*N*) was calculated assuming an occurrence rate (*P*) of the outcome variable of 70% (9), with a margin of error (*ε*) set at 0.1P and a 95% confidence level (*Z* = 1.96), yielding a minimum sample size of 336. Accounting for an anticipated response rate of 70%, the final target sample size was approximately 480. The actual sample size of 530 in this study met this requirement. A total of 583 questionnaires were distributed via the platform, and 530 valid questionnaires were collected, resulting in a valid response rate of 90.91%.

### 2.3 Quality control

To ensure the independence of the questionnaires, the online platform was configured to allow only one submission per IP address or device. Additionally, questionnaires with a completion time of less than 200 s were excluded during the backend data screening process, as were those with incorrect responses to validation questions embedded to ensure attentive participation.

### 2.4 Definitions

The 2017 World Health Organization (WHO) guideline on managing advanced HIV disease and rapid initiation of antiretroviral therapy recommends that all individuals diagnosed with HIV should start ART within 7 days, defined as rapid initiation of ART, with same-day initiation recommended where possible (5). However, specific recommendations regarding the timing of ART initiation vary across authoritative guidelines. Therefore, based on the specific treatment landscape for PLWHA in Lanzhou, this study defines rapid initiation of ART as starting treatment within 30 days of diagnosis. The rapid initiation rate was calculated as follows: (Number of patients who received rapid ART / Total number of patients receiving treatment) × 100%. Material support refers to the provision of tangible assistance, such as meals, daily necessities, and medications, to infected individuals.

### 2.5 Statistical analysis

Data were analyzed using SPSS 27.0. Using whether PLWHA underwent rapid ART initiation as the dependent variable, all variables with *p* < 0.1 in the univariable analysis were included in a multivariable logistic regression model. Variable selection was performed using the stepwise regression method (*α* = 0.05 for entry, *β* = 0.10 for removal). ORs and their 95% CIs were calculated. Categorical data were described as percentages (%), and comparisons between two groups were performed using the *χ*^2^ test. A *p*-value < 0.05 was considered statistically significant.

## 3 Results

### 3.1 Baseline characteristics

A total of 530 participants were included, of whom 506 (95.47%) were male. The majority (268, 50.57%) were in the 31 to <46 age group. 481 participants (90.75%) were of Han ethnicity, and 303 (57.17%) had an educational level of college or above. Most participants were unmarried (283, 53.40%), and the largest occupational group was flexible employment (269, 50.75%). The monthly income for most participants (333, 62.83%) was below 5,000 CNY. The infection status was known to spouses/family members for 368 participants (69.44%), and 207 (39.06%) reported having peers living with HIV. Univariable analysis (0 = underwent rapid ART initiation, 1 = did not undergo rapid ART initiation) revealed that age, educational level, cross-regional treatment, marital status, monthly income, whether spouses/family members were aware of the infection status, and the presence of infected peers were associated with rapid ART initiation (*p* < 0.1), as shown in Table 1.

**Table 1:** Baseline demographic characteristics and univariable analysis.

Variable	Proportion of cases(%)	Rapid ART(%)	Non-rapid ART(%)	Univariable analysis		
				*χ* ^2^	*P*	*OR*(95%CI)
Gender				0.276	0.599	
Male	506(95.47)	399(78.85)	107(21.15)			1.00
Female	24(4.53)	20(83.33)	4(16.67)			0.75(0.25–2.23)
Age (years)				8.856	0.031	
≥61	27(5.09)	15(55.56)	12(44.44)			1.00
15 ~ <31	79(14.91)	65(82.28)	14(17.72)			0.27(0.10–0.70)
31 ~ <46	268(50.57)	215(80.22)	53(19.78)			0.31(0.14–0.70)
46 ~ <61	156(29.43)	124(79.49)	32(20.51)			0.32(0.14–0.76)
Ethnic group				2.382	0.123	
Han Chinese	481(90.75)	376(78.17)	105(21.83)			1.00
Others	49(9.25)	43(87.76)	6(12.24)			0.50(0.21–1.21)
Level of education				5.023	0.081	
Primary school and below	29(5.47)	27(93.10)	2(6.90)			1.00
Junior high school, senior high school, or technical secondary school	198(37.36)	161(81.31)	37(18.69)			3.10(0.71–13.63)
Junior college degree or above	303(57.17)	231(76.24)	72(23.76)			4.21(0.98–18.13)
Cross-regional treatment				5.491	0.064	
District and county level	203(38.30)	150(73.89)	53(26.11)			1.00
Inter-district/county areas within the city	262(49.43)	217(82.82)	45(17.18)			0.59(0.38–0.92)
Across cities and prefectures within the province	63(11.89)	50(79.36)	13(20.64)			0.71(0.36–1.40)
Marital status				10.133	0.006	
Married	172(32.45)	150(87.21)	22(12.79)			1.00
Unmarried	283(53.40)	211(74.56)	72(25.44)			2.33(1.38–3.92)
Divorced or widowed	75(14.15)	58(77.33)	17(22.67)			2.00(0.99–4.03)
Career				4.536	0.209	
Stable employment group*	121(22.84)	90(74.38)	31(25.62)			1.00
Flexible employment group*	269(50.75)	217(80.67)	52(19.33)			0.70(0.42–1.16)
Agriculture and the working population	74(13.96)	63(85.14)	11(14.86)			0.51(0.24–1.08)
Non-employed population	66(12.45)	49(74.24)	17(25.76)			1.00(0.51–2.00)
Monthly income				5.067	0.024	
Under 5,000 yuan^^a^	333(62.83)	253(75.98)	80(24.02)			1.00
Over 5,000 yuan^^a^	197(37.17)	166(84.26)	31(15.74)			0.59(0.37–0.93)
Whether the spouse/family is aware of the infection status				9.001	0.003	
No	162(30.56)	115(70.99)	47(29.01)			1.00
Yes	368(69.44)	304(82.61)	64(17.39)			0.52(0.33–0.80)
Whether there are infected individuals among those around me				5.067	0.024	
No	323(60.94)	245(75.85)	78(24.15)			1.00
Yes	207(39.06)	174(84.06)	33(15.94)			0.60(0.38–0.94)

According to the latest foreign exchange market rates, 1 CNY ≈ 0.1376 USD, therefore 5,000 CNY ≈ 688 USD.

### 3.2 Multivariable analysis

Variables significant in the univariable analysis were included in a multivariable logistic regression model. The results demonstrated that ages 15 to <31 years (OR = 0.21, 95% CI = 0.07–0.60), 31 to <46 years (OR = 0.28, 95% CI = 0.11–0.68), cross-district treatment within the city (OR = 0.57, 95% CI = 0.36–0.92), monthly income ≥5,000 CNY (OR = 0.60, 95% CI = 0.37–0.99), disclosure of infection status to spouses/family members (OR = 0.60, 95% CI = 0.38–0.96), and the presence of infected peers (OR = 0.55, 95% CI = 0.34–0.88) were facilitating factors for rapid ART initiation among PLWHA. Conversely, being unmarried (OR = 2.52, 95% CI = 1.38–4.61) was identified as a barrier to rapid ART initiation, as detailed in Table 2.

**Table 2:** Multivariable analysis of factors influencing rapid ART initiation.

Variable		*β*	SE	Wald χ^2^	*P*	OR(95%CI)
Age (years) ^^a^	15 ~ <31	−1.555	0.533	8.508	0.004	0.21(0.07–0.60)
	31 ~ <46	−1.285	0.459	7.826	0.005	0.28(0.11–0.68)
	46 ~ <61	−0.905	0.468	3.744	0.053	0.40(0.16–1.01)
Educational level ^^b^	Junior high school, senior high school, or technical secondary school	1.182	0.773	2.340	0.126	3.26(0.72–14.84)
	Junior college degree or above	1.481	0.771	3.686	0.550	4.40(0.97–19.92)
Cross-regional treatment^^c^	Urban areas spanning multiple districts and counties	−0.557	0.243	5.250	0.022	0.57(0.36–0.92)
	Across different cities and prefectures within the province	−0.263	0.364	0.523	0.470	0.77(0.38–1.57)
Marital status^^d^	unmarried	0.925	0.308	9.021	0.003	2.52(1.38–4.61)
	Divorced or widowed	0.686	0.386	3.162	0.075	1.99(0.93–4.23)
Monthly income^^e^	Over 5,000 yuan	−0.504	0.253	3.982	0.046	0.60(0.37–0.99)
Whether the spouse/family is aware of the infection status^^f^	Yes	−0.509	0.241	4.470	0.034	0.60(0.38–0.96)
Whether there are infected individuals among those around me^^g^	Yes	−0.606	0.246	6.088	0.014	0.55(0.34–0.88)

^^a^Reference group for Age is ≥61 years; ^^b^Reference group for Education level is primary school or below; ^^c^Reference group for Cross-district treatment is within the county/district; ^^d^Reference group for Marital status is married; ^^e^Reference group for Monthly income is below 5,000 CNY (≈688 USD); ^^f^Reference group for Disclosure to spouse/family is no; ^^g^Reference group for Having infected peers is no.

### 3.3 Association between social support and rapid ART initiation decisions

Among the 368 participants whose infection status was known to spouses/family members, 82 (22.28%) reported a positive attitude from them after disclosure, while 286 (77.72%) reported a neutral or negative attitude. The difference in the rapid ART initiation rate based on the attitude of spouses/family members after disclosure was statistically significant (*χ*^2^ = 4.281, *p* = 0.039). Of those whose status was known, support was provided by spouses/family members in 313 cases (85.05%), while no support was provided in 55 cases (14.95%). The difference in the rapid ART initiation rate based on whether support was provided by spouses/family members was statistically significant (*χ*^2^ = 4.281, *p* = 0.039). Furthermore, among the 207 PLWHA who had peers living with HIV, support was provided by peers in 176 cases (85.02%), while no support was provided in 31 cases (14.98%). The difference in the rapid ART initiation rate based on whether support was provided by peers was not statistically significant (*p* = 0.058). See Table 3 for details.

**Table 3:** Comparison of rapid ART initiation rates by social support status.

Group		Proportion of cases(%)	Rapid ART(%)	Non-rapid ART(%)	*χ* ^2^	*P*
The attitude of the spouse/family members upon being informed	Proactive	82(22.28)	74(90.24)	8(9.76)	4.281	0.039
	Neutral/Passive	286(77.72)	230(80.42)	56(19.58)		
Does one’s spouse or family provide support	No	55(14.95)	40(72.73)	15(27.27)	4.395	0.036
	Yes	313(85.05)	264(84.35)	49(15.65)		
Does one’s companion provide support	No	31(14.98)	22(70.97)	9(29.03)	–	0.058^^a^
	Yes	176(85.02)	152(86.36)	24(13.64)		

^^a^Fisher’s Exact Test was used because more than 20% of the cells had an expected count of less than 5.

### 3.4 Specific types of social support received

Among the support provided by spouses/family members, psychological support was the most common, accounting for 62.5% of this subgroup, and it was associated with the highest rapid ART initiation rate of 51.6%. The proportions of financial support and material support were similar, both approximately 38–39%, and their corresponding rapid initiation rates were also comparable, at around 33%. Among the 55 cases who received no support, the rapid initiation rate was the lowest at only 10.9%, which was significantly lower than the rates observed with any form of support. Similar results were observed in the subgroup with infected peers: psychological support had the highest proportion (69.57%) and the greatest rapid ART initiation rate (57.97%), while the absence of peer support was associated with the lowest proportion (14.98%) and the smallest rapid initiation rate (10.63%). See Table 4.

**Table 4:** Relationship between different types of social support and rapid ART initiation.

Source and type of support	Number of cases (*n*)	Proportion (%)	Rapid initiation (*n*)	Non-rapid initiation (*n*)	Rapid ART initiation rate (%)
Spouse/family^^a^ (*n* = 368)					
Financial Support	141	38.32	125	16	33.97
Psychological Support	230	62.50	190	40	51.63
Material Support	144	39.13	120	24	32.61
Other	63	17.12	56	7	15.22
No Support	55	14.95	40	15	10.87
Peers^^a^ (*n* = 207)					
Financial Support	38	18.36	37	1	17.87
Psychological Support	144	69.57	120	24	57.97
Material Support	34	16.43	31	3	14.98
Other	26	12.56	23	3	11.11
No Support	31	14.98	22	9	10.63

(1) ^^a^This was a multiple-response item, therefore the sum of the response proportions exceeds 100%. (2) The proportions of support types and rapid ART initiation rates in this table were calculated using “PLWHA whose family members were aware of their status (*n* = 368)” and “PLWHA who had infected peers (*n* = 207)” as the respective denominators. Data under the same source (spouse/family or peers) are comparable.

## 4 Discussion

Rapid ART has been proven to significantly improve treatment adherence and viral suppression rates among PLWHA, while effectively reducing the risk of delayed treatment (10). However, in clinical practice, patients often delay seeking treatment due to lack of information, psychological conflicts, and environmental factors, leading to initiation times that far exceed internationally recommended timelines (11). Social support is recognized as a key psychosocial factor influencing the acceptance of rapid ART initiation. Studies indicate that awareness and support from spouses/family members and peers can significantly enhance patients’ disease awareness and treatment acceptance, thereby reducing anxiety, strengthening treatment confidence, and ultimately facilitating quicker initiation of ART (12, 13).

The univariable and multivariable analysis results in this study indicated that compared to patients aged ≥61 years, those aged 15 ~ <31 years and 31 ~ <46 years were less likely to delay ART initiation beyond 30 days after diagnosis. It is noteworthy that, in terms of the statistical effect size, the 15 ~ <31 age group (OR = 0.21) had the strongest magnitude of effect in promoting rapid ART initiation. A possible reason is that PLWHA aged 15 ~ <31 are often more adept at accessing up-to-date information on HIV prevention and treatment through the internet and social media. Consequently, they are more familiar with modern treatment concepts like “rapid initiation,” making them more likely to start antiviral therapy promptly (14). PLWHA in the 31 ~ <46 age group often have stable families and social circles, making it easier for them to receive psychological and material support for treatment from family and friends, which in turn facilitates quicker acceptance of ART (15). Furthermore, older patients often have multiple comorbidities, such as hypertension and diabetes. Therefore, before initiating ART, physicians typically need to assess potential drug interactions and adverse reactions in conjunction with their regular medications and often wait until the patient’s clinical indicators are controlled within a general range before starting treatment (16). Finally, older patients often bear a higher proportion of out-of-pocket payments for medications, and economic constraints may also lead them to delay seeking treatment (17).

Compared to PLWHA receiving treatment within the same county/district as their residence, those who sought treatment across districts/counties were less likely to delay ART initiation beyond 30 days after diagnosis. This finding appears contrary to the common understanding that geographical distance poses a barrier to treatment (18). This result may stem from limitations in the study methodology: the respondents were all patients who had been on long-term, standardized treatment. Their recollection of their residence at the time of initial diagnosis might be subject to recall bias. Furthermore, the data did not include PLWHA who were lost to follow-up or never initiated treatment due to geographical barriers. Therefore, the “cross-district/county” group in this study might actually represent individuals who overcame initial barriers and possessed a stronger motivation for treatment, thus leading to results that appear contrary to theoretical expectations.

Unmarried patients had 2.52 times the odds of not receiving rapid ART compared to married patients, meaning they were more likely to initiate ART beyond 30 days. A possible explanation is that married patients often receive emotional, financial, material, and psychological support from their partners, whereas unmarried patients generally lack such support, potentially leading to delays in seeking treatment (19).

Patients with a monthly income ≥5,000 CNY had 0.60 times the odds of not receiving rapid ART compared to those earning <5,000 CNY, indicating that higher-income patients were less likely to delay initiation beyond 30 days. This may be because low-income groups are often more concerned about the economic burden of the disease, leading to postponed care.

Furthermore, this study found that patients whose infection status was known to spouses/family members and those who had peers living with HIV had 0.60 and 0.55 times the odds, respectively, of not receiving rapid ART compared to their counterparts. This suggests that disclosure to spouses/family and having infected peers facilitated earlier ART initiation. Research indicates that disclosure creates a “spillover effect”; even if patients delay seeking care, they may adjust their health behaviors based on their family’s awareness, thereby initiating ART sooner (20). The presence of infected peers is associated with lower levels of HIV-related stigma and higher disease treatment awareness and acceptance, making patients more proactive in seeking treatment (21).

Among the 530 participants included in this study, 368 had disclosed their infection status to spouses/family members. Of these, only 82 participants reported a positive attitude from their spouses/family members after disclosure, and the rapid ART initiation rate was significantly higher in this group compared to those who received a neutral or negative attitude. Studies indicate that a positive attitude from spouses/family members after disclosure can provide emotional comfort, daily life assistance, and treatment adherence encouragement, thereby facilitating quicker ART initiation (22), which is consistent with the findings of this study. Furthermore, among the spouses/family members aware of the participants’ status, 313 provided one or multiple forms of support, including financial, psychological, material, and other types of assistance. The rapid ART initiation rate was significantly higher among those who received support compared to those who received no support. This may be because support from spouses/family members alleviates anxiety and depression, enhances self-acceptance, and strengthens treatment confidence. Financial support can cover expenses related to examinations, medications, and transportation. Multiple studies have identified treatment costs as a key factor contributing to delayed treatment initiation (23–25). Additionally, spouses/family members can access disease-related treatment and prevention information through various professional channels, helping patients gain a quicker and deeper understanding of the disease progression and treatment options. This reduces the time required for patients to acquire information independently and accelerates ART initiation. Accurate information also helps correct misconceptions promptly, preventing treatment delays due to misinformation.

Among the 530 participants, 207 reported having peers living with HIV. Of these, 176 received one or multiple forms of support from their peers, including financial, psychological, material, and other assistance. However, the difference in the rapid ART initiation rate based on whether peer support was provided was not statistically significant. This result contrasts sharply with the significant difference observed regarding support from spouses/family members. Support from spouses/family members often involves deeper emotional bonds, shared daily lives, and common economic responsibilities. Therefore, during the initial diagnosis period, when patients face significant psychological barriers, support from spouses/family members provides a sense of “security,” profoundly influencing their decision to initiate ART promptly (26). In contrast, support from peers primarily involves resource sharing, experience exchange, and mutual encouragement during long-term treatment, which may have less impact on the initial treatment decision compared to support from spouses/family members. Moreover, some studies categorize family support as part of the “microsystem,” while peer and community support are classified under the “mesosystem” or “exosystem.” These studies confirm that the direct influence of the family microsystem is stronger than that of the peer exosystem (27, 28), which strongly validates the contrasting impacts on rapid ART initiation observed between spousal/family support and peer support in this study.

This study also found that, whether from spouses/family or peers, receiving any form of support was associated with a higher likelihood of rapid ART initiation compared to receiving no support. The rapid initiation rates in the groups receiving no support from spouses/family and peers were 10.87 and 10.63%, respectively, both lower than the rates observed with any other type of support. This finding is consistent with the results reported by K. Rivet Amico et al. (29). Furthermore, among the different types of support provided by either spouses/family or peers, psychological support was associated with the highest rapid initiation rate. This may be related to patients’ increased willingness to accept immediate treatment after receiving emotional comfort and reduced anxiety (30). Studies have shown that PLWHA who receive emotional support often exhibit lower risks of depression, anxiety, and post-traumatic stress disorder, making them more inclined to initiate antiretroviral therapy on the day of diagnosis or the following day, thereby validating the above conclusion (31). In summary, psychological support at the emotional level is a crucial factor in promoting rapid ART initiation among PLWHA and deserves emphasis in clinical interventions and public health programs.

In summary, besides demographic factors such as ages 15 ~ <31 years, 31 ~ <46 years, cross-district treatment within the city, and monthly income ≥5,000 CNY, which promote rapid ART initiation, social factors—including support from spouses/family members and peers—are also crucial. Therefore, integrating social factors into a comprehensive intervention framework for rapid ART initiation is essential. Establishing a collaborative support network involving families, peers, and healthcare facilities can enhance patients’ willingness and timeliness to start treatment, ultimately improving treatment outcomes for PLWHA.

Finally, the main limitations of this study originate from the following: the HIV epidemic in the study region is predominantly among males; the questionnaire samples were collected using a convenience sampling method. Women may have higher sensitivity regarding their personal information when faced with the option of completing the questionnaire, leading to a relatively lower proportion of female samples collected. Consequently, the overall gender ratio in the questionnaire sample was considerably unbalanced. Based on this, the generalizability of the findings may be more applicable to male PLWHA.

## Data Availability

The original contributions presented in the study are included in the article/supplementary material, further inquiries can be directed to the corresponding authors.
